# New molecular structure based models for estimation of the CO_2_ solubility in different choline chloride-based deep eutectic solvents (DESs)

**DOI:** 10.1038/s41598-023-35747-8

**Published:** 2023-05-25

**Authors:** Farnoosh Dehkordi, Mohammad Amin Sobati, Ali Ebrahimpoor Gorji

**Affiliations:** School of Chemical Engineering, Iran University of Science and Technology (IUST), Tehran, Iran

**Keywords:** Cheminformatics, Green chemistry, Chemical engineering, Computational science, Statistics

## Abstract

In this study, CO_2_ solubility in different choline chloride-based deep eutectic solvents (DESs) has been investigated using the Quantitative Structure–Property Relationship (QSPR). In this regard, the effect of different structures of the hydrogen bond donor (HBD) in choline chloride (ChCl) based deep eutectic solvents (DESs) has been studied in different temperatures and different molar ratios of ChCl as hydrogen bond acceptor (HBA) to HBD. 12 different datasets with 390 data on the CO_2_ solubility were chosen from the literature for the model development. Eight predictive models, which contain the pressure and one structural descriptor, have been developed at the fixed temperature (i.e. 293, 303, 313, or 323 K), and the constant molar ratio of ChCl to HBD equal to 1:3 or 1:4. Moreover, two models were also introduced, which considered the effects of pressure, temperature, and HBD structures, simultaneously in the molar ratios equal to 1:3 or 1:4. Two additional datasets were used only for the further external validation of these two models at new temperatures, pressures, and HBD structures. It was identified that CO_2_ solubility depends on the “EEig02d” descriptor of HBD. “EEig02d” is a molecular descriptor derived from the edge adjacency matrix of a molecule that is weighted by dipole moments. This descriptor is also related to the molar volume of the structure. The statistical evaluation of the proposed models for the unfixed and fixed temperature datasets confirmed the validity of the developed models.

## Introduction

The significant emission of greenhouse gases like CO_2_ has led to two significant global challenges, which are called “Global Warming” and “Climate Change”^1^. During the last decade, the presence of CO_2_ gas in the atmosphere has exceeded the acceptable limits (i.e. 350 ppm)^2–4^. Hence, an extensive effort is required to eliminate CO_2_ gas from the atmosphere. There are some advanced technologies for decreasing CO_2_ emission, like Carbon Capture and Storage (CCS). CCS technologies are mainly categorized into three groups: pre-combustion, post-combustion (PCC), and Oxy-combustion (oxy-fuel)^5^. Among these methods, the PCC method is more practical and economical. It is still necessary to solve several economic, technological, environmental, and safety challenges such as (i) improving the efficiency of CO_2_ capture, (ii) reducing the costs of the process, and (iii) ensuring CO_2_ storage is environmentally sustainable^6^. Applying aqueous Alkanolamine solvents (e.g. MEA) in the PCC method is conventional because of its high reactivity with CO_2_, availability, low cost, and low viscosity. However, there are still several flaws in using such kinds of solvents, including high loss of the solvent, degradation, corrosion, high energy consumption during the regeneration process, environmental problems, and high regeneration costs^7–9^. As a result, it is essential to develop new green and cheap solvents for CO_2_ capture processes.

Research in recent years have increasingly focused on the development of novel solvents such as Ionic Liquids (ILs), and Deep Eutectic Solvents (DESs) to replace the conventional volatile organic compounds (VOC) in different chemical and industrial processes^10–12^.

Compared to conventional CO_2_ capture solvents (i.e., amines), ILs are more capable due to their attractive intrinsic properties such as low volatility, high thermal stability, and excellent CO_2_ solubility^13,14^. It is well known that ILs are efficient physical sorbents of CO_2_, that their specifications can be tuned by selecting the proper cations and anions. Despite these advantages, using ILs for CO_2_ capture in industrial applications has several drawbacks, including their high viscosity, complicated and expensive synthesis and purification processes, and high cost. A growing concern exists regarding the toxicity of several ILs^15^. There are new classes of solvents known as DESs, which have additional merits of low cost, low toxicity, biodegradability, easy preparation, and no need to purification^16^. The DESs can be synthesized by mixing a hydrogen bond donor (HBD) (e.g. carboxylic acids, amides, amines, alcohol, or metal halides) with a hydrogen bond acceptor (HBA) (e.g. quaternary phosphonium or ammonium salts) in the appropriate molar ratios^17^. The most promising property of DESs is the diversity of structures. Because of their inherent benefits, including low vapor pressure, high thermal and chemical stability, non-flammability, and a wide range of adjustability, DESs have gained considerable attention^18,19^. In particular, choline-based DESs have been investigated intensively. Since the choline-based DESs are mainly constituted natural compounds; therefore, they have no harmful environmental influences. Among the widely used choline salts, choline chloride (ChCl) is a non-toxic, biodegradable, and inexpensive material either synthesized from product or by-product from fossil reserves(i.e. petroleum) or extracted from the biomass^19^.

The theoretical and experimental studies of DESs have been conducted in different applications such as CO_2_ capture^20^, desulfurization^21,22^, and separation process^23^. There are several experimental studies in the literature on the measurement of the CO_2_ solubility in different types of DESs.

In the first experimental study, Li et al.^24^ examined ChCl-based DESs as CO_2_ absorbents at various pressures (0.85 − 12.52 MPa), temperatures (313.15 − 333.15 K), and molar ratios (1:1.5, 1:2, or 1:2.5). Their results demonstrated that the CO_2_ solubility decreased by increasing temperature and increased by increasing pressure. Besides, it was confirmed that the molar ratio has a great effect on the CO_2_ solubility in DESs. Therefore, the ChCl/Urea (1:2) (the so-called reline system) indicated a higher CO_2_ solubility value compared to other DESs from a ChCl/Urea molar ratio of 1:1.5 and 1:2.5. Li et al.^25^ also studied a series of ChCl-based DESs, including ChCl/Phenol (1:2, 1:3, 1:4), ChCl/Triethylene glycol (1:3 and 1:4) and ChCl/Diethylene glycol (1:3 and 1:4). It was found that the solubility of CO_2_ in ChCl/Triethylene glycol (1:4) is the highest compared to other DESs. In another work, Leron et al.^26^ measured the solubility of CO_2_ in the reline system at the expanded temperature range of 303.15 to 343.15 K. Leron et al.^27,28^ also reported the CO_2_ solubility in DESs containing ChCl/Glycerol (1:2) or ChCl/Ethylene glycol (1:2) and presented higher CO_2_ solubility compared to the imidazolium-based ILs. Sarmad et al.^20^ reported 209 data points on the CO_2_ solubility in 35 different DESs at 298.15 K and pressure lower than 2 MPa. Chen et al.^29^ studied the CO_2_ solubility in the ChCl/1,2-Propanediol, 1,4-Butanedioland, and 2,3-Butanediol (1:3 and 1:4). Their results approved that ChCl/2,3-Butanediol (1:4) had the highest capacity of CO_2_ absorption. Lu et al.^30^ investigated the CO_2_ solubility in the ChCl/Levulinic acid or ChCl/Furfuryl alcohol (1:3, 1:4, and 1:5). According to their results, ChCl/Levulinic acid (1:5) indicated a higher capacity of CO_2_ absorption compared to Furfuryl alcohol. Therefore, it can be concluded that the presence of a specific HBD along with a fixed HBA (i.e., ChCl) can influence the CO_2_ solubility in DES significantly. It was verified that the CO_2_ solubility in DESs depends on the type of HBD and HBA, the HBA to HBD molar ratio, viscosity, and water content of DESs, and operating pressure and temperature^31^.

It should be mentioned that numerous DESs can be synthesized by combining different HBA and HBD. It is challenging to select the most suitable DESs for the CO_2_ capture processes based on the experimental studies. Therefore, an efficient theoretical method is needed to predict the CO_2_ solubility in DESs.

One of the most popular methods of Machine Learning (ML) to complement experimental, analytical techniques is the Quantitative Structure–Property Relationship (QSPR). To develop QSAR/QSPR models, the chemical structures are fragmented into structural groups, and mathematical algorithms are applied to the data. The general idea is to derive an expression in Property = f (X_1_, X_2_, X_3_, …X_n_), where each variable can be a chemical structure feature (i.e. molecular descriptors) or physicochemical property. The derived function can significantly help to gain the deeper molecular insights into the relationships between the process-relevant properties of molecules and predict property relationships for new but related materials, and also help to explain the measured characteristics^32^.

Lemaoui et al. presented new QSPR models to predict the viscosity, density, and electrical conductivity of DESs by a multilinear regression (MLR) analysis. Their results confirmed that the developed models for the studied DESs properties were able to predict the density, viscosity, and electrical conductivity of the DESs with a satisfactory accuracy (i.e. R^2^ values of 0.9839, 0.9874, and 0.985, respectively)^33,34^. Balali et al.^22^ presented QSPR models to take into account the effect of HBD structures on the thiophene distribution (β_2_) between hydrocarbon phases and ChCl-based DESs in the ternary systems. Table S1 in the supporting information file shows the available QSPR models in the literature for the prediction of different properties of DESs.

Numerous studies have been reported in the literature for predicting the CO_2_ solubility in DESs using different approaches. The CO_2_ solubility in choline chloride-based DESs has been predicted successfully using thermodynamic modeling approaches such as modified Peng-Robinson (PR) EoS^35,36^, density functional theory (DFT)^37,38^, and molecular dynamics (MD) simulation^38–40^. Zubeir et al.^41^ applied the Perturbed-Chain Statistical Associating Fluid Theory (PC-SAFT) to express the CO_2_ solubility in DESs in the pressures up to 2 MPa and temperature ranges 298.15 and 318.5 K using two pseudo-pure components and strategies of individual-component. Kamgar et al.^42^ employed COSMO-RS and NRTL models to predict the solubility of CO_2_, CH_4_, CO, N_2_, and H_2_ in the reline. Their results indicated that the models could only predict the solubility at high temperatures and low pressures. Recently, Alioui et al.^43^ have combined the MD methods and COSMO-RS to study the solubility of CO_2_ in seven ChCl and phosphonium-based DESs. Liu et al.^44^ assessed COSMO-RS to predict the CO_2_ solubility and Henry’s constants of CO_2_ in DESs based on the experimental data. Different thermodynamic methods developed for the solubility of CO_2_ in different DESs are summarized in Table S2 in the supporting information file.

As can be observed in Table S2, few QSPR models have been developed to predict the CO_2_ solubility in DESs.

In the first QSPR study on the prediction of CO_2_ solubility in DESs, Wang et al.^45^ developed both linear and non-linear models using COSMO-RS-derived descriptors of HBA and HBD structures, temperature, pressure and molar ratio of HBA to HBD. Aside from the numerous benefits of their work, a few drawbacks were also observed. Although they used large number of descriptors for each component of DESs (i.e. HBA and HBD), their developed linear model have limited prediction capability. Besides, their developed model was not descriptive due to the application of sigma profile descriptors, which are not interpretable. Furthermore, they used molar ratio as an independent variable in their linear model. The relationship between HBA to HBD molar ratio and solubility is not linear (see Fig. S1 in the supporting information file). Therefore, in the present study, it was tried to find the most important interpretable descriptor of HBD in the presence of the fixed HBA (i.e. Choline chloride).

Kumar et al.^46^ presented 12 QSPR models for the prediction of the CO_2_ capture capacity of DESs considering the effect of HBA and HBD structures, HBA to HBD molar ratio, temperature and pressure. The Monte Carlo method was used to determine the appropriate coefficients of each quasi-SMILES descriptors for 72 different DESs (including 19 different HBAs and 20 different HBDs). Their developed models included four random splits from datasets as well as three target functions with and without criterion of the predictive potential examination (i.e. index of ideality of correlation (IIC) and correlation intensity index (CII)). Then, they introduced the model with the highest accuracy according to different statistical parameters. Although their work was very comprehensive and valuable because of using diverse dataset and also high prediction accuracy of model, it seems that the parameters of their model cannot be interpreted and the effect of each parameter in the CO_2_ absorption mechanism cannot be investigated. In other words, it seems that they paid more attention to the predictability of the model instead of describing why and how each of the variables in the developed model affect the CO_2_ capture capacity. Therefore, in the present study, it has been tried to develop descriptive and predictive QSPR models with meaningful and interpretable descriptor.

Halder et al.^47^ performed multicriteria decision techniques to develop multi-objective models to investigate two properties (i.e. viscosity, and CO_2_ uptake capacity), simultaneously. Their work is valuable because the viscosity of DESs plays a significant role in the final solvent choice. They developed two linear QSPR models for predicting the CO_2_ uptake capacity and viscosity of DESs, separately. Then, they used the Derringer’s desirability function to integrate these two models for identification of the DESs with high CO_2_ absorption capacity and low viscosity. Although their work was very innovative and comprehensive, there are few flaws in their work. First of all, according to the MD simulation performed by Alizadeh et al.^48^, there is a strong effect of HBD structures and anion part of HBA and a slight effect of cation part of HBA on CO_2_ solubility in the DESs. Meanwhile, at a lower pressure, the HBD–CO_2_ interaction is dominant, and at a higher pressure, it is the anion–CO_2_ interaction. In another word, HBD structures have a greater effect on the CO_2_ absorption at low pressures and HBA structures at high pressures. However, Halder et al.^47^ have considered the effectiveness of HBA (both cation and anion parts) and HBD in all conditions to be the same. Second, temperature and pressure variables were not present in their model and the prediction was made only by structural variables. While, it has been proven that temperature and pressure have a significant effect on the CO_2_ absorption. Thus, in the present study, an effort has been made to investigate the effect of HBD structures on the CO_2_ solubility in low pressure (i.e. physical absorption) while considering the key parameters of temperature and pressure in the developed model. Therefore, in this study, it was tried to fill the observed gaps in the recent invaluable researches.

In this study, the QSPR method is applied as a robust tool to develop predictive models for solubility of CO_2_ in the DESs with a fixed HBA (i.e. ChCl) with the molar ratio of HBA to HBD equal to 1:3 and 1:4. At first, some QSPR models are developed, which can consider the effect of the HBD structures and pressure at fixed temperature (i.e. 293, 303, 313, or 323 K). Then, the CO_2_ solubility dependence on temperature was considered along with the pressure and HBD descriptor. This approach can efficiently predict the CO_2_ solubility for new ChCl-based DESs in new temperatures. Moreover, two additional datasets were applied for further external validation to confirm the robustness of the unfixed temperature models.

## The QSPR method

### Dataset

The available experimental data of CO_2_ solubility in ChCl-based DESs with molar ratios of 1:3 and 1:4 were collected from the literature, at first. The range of P, T, and CO_2_ solubility for each dataset was shown in Table 1. The total number of CO_2_ solubility data points is 390. As can be seen in Table 1, the variation of the involved HBD in DESs was nine. In the present study, the values of CO_2_ solubility (x: mole of CO_2_ per mole of DES) have been converted into the form of the natural logarithm (i.e., ln(x)) for the model development. A common technique used to ensure the reliability of the developed QSPR models is to divide the datasets into two separate sets called “train” and “test”. It should be mentioned that the QSPR model was developed using the train set, and the internal validation technique can be applied to this set. The developed QSPR model should be externally validated by taking some HBDs out of the datasets and putting them into the test set. Through this work, the prediction capability and accuracy of the developed model can be assessed. In order to increase the robustness of the external validation, it was tried to select the test set in such a way to consist of some HBD structures, which are different from the involved structures in the train set. In addition, datasets no (11) and (12) have been used for further external validation of the developed models in the unfixed temperature status and applied the models at new temperatures, pressures and HBD structures. Furthermore, the applicability domain of the constructed models has also been checked for both the train and test sets, which indicates that both of them contain DESs with considerable differences from a molecular structure viewpoint.

**Table 1:** The variation ranges of pressure and solubility for each studied dataset in the present study.

No. of dataset	T^a^	Molar ratio (ChCl:HBD)	No. of involved HBD	Pressure range^b^	Solubility range^c^	No. of data	Refs
1	293–323	1:3	9^d^	0.515–5.853	0.0014–0.0311	181	^25,29,30,49,50^
2	293–323	1:4	9	0.521–5.842	0.0014–0.0326	181	^25,29,30,49,50^
3	293	1:3	7^e^	0.515–5.294	0.0021–0.0282	36	^25,29,50^
4	293	1:4	7	0.521–5.26	0.0022–0.0288	36	^25,29,50^
5	303	1:3	9	0.553–5.828	0.002–0.0311	49	^25,29,30,49,50^
6	303	1:4	9	0.725–5.815	0.0027–0.0326	49	^25,29,30,49,50^
7	313	1:3	9	0.575–5.817	0.0017–0.0266	48	^25,29,30,50^
8	313	1:4	9	0.534–5.688	0.0014–0.0281	48	^25,29,30,50^
9	323	1:3	9	0.631–5.853	0.0014–0.0212	48	^25,29,30,50^
10	323	1:4	9	0.547–5.842	0.0014–0.0234	48	^25,29,30,50^
11	298,333	1:3	3f.	0.859–10	0.0018–0.0454	13	^30,35^
12	298,333	1:4	5^ g^	0.826–10	0.0022–0.0419	15	^30,35^

^a^T is the temperature in units of K.

^b^variation domain of P for each HBD. P is the pressure in units of bar.

^c^variation domain of x for each HBD. x is the solubility of CO_2_ in DES in units of mole CO_2_/mole DES.

^d^1,2-Propanediol, 1,4-Butanediol, 2,3-Butanediol, Diethylene glycol, Guaiacol, Phenol, Triethylene glycol, Furfuryl alcohol, Levulinic acid.

^e^1,2-Propanediol, 1,4-Butanediol, 2,3-Butanediol, Diethylene glycol, Guaiacol, Phenol, Triethylene glycol.

^f^Furfuryl alcohol, Levulinic acid, glycerol.

^g^Triethylene glycol, Ethylene glycol, Furfuryl alcohol, Levulinic acid, Urea.

### Optimization of HBD structures and descriptors calculation

Before calculating the descriptors of each HBD, it is essential to optimize their molecular structures. The 3D structures of 9 HBD molecules were drawn using gauss-view software^51^ and then were submitted to geometry optimization using the density functional theory (DFT) at the level of B3LYP and 6–31 + G (d,p)^52^. Afterward, Dragon software^53^ was applied to calculate the different kinds of 1D, 2D, and 3D descriptors. In order to reduce the number of computed descriptors (i.e. 3224), constant and semi-constant descriptors, and the descriptors with high intercorrelation (> 98%) were eliminated. Therefore, the remaining 444 molecular descriptors of the HBD structures were used for the model construction.

### Basic theory and model construction procedure

#### Basic theory

CO_2_ solubility in the gas–liquid systems (i.e. CO_2_ in DES) is defined as follows:

1 $$x=\frac{mole\, {CO}_{2}}{mole\, DES}.$$

According to Li et al.^24^, the CO_2_ solubility is dependent on the temperature and pressure as well as the HBA to HBD molar ratio.

In a constant HBA to HBD molar ratio, the relationship between ln(x) and ln(P) can be considered as follows (see Fig. S2 in the supplementary file):

2 $$\mathrm{ln}\left(x\right)=a\times \mathrm{ln}\left(P\right)+b,$$

where a, and b represent the adjustable parameters. As it is clear, the molecular structure of HBDs can play a key role in different processes such as desulfurization^22^ and CO_2_ solubility^20^. In this study, the QSPR method will be used to correlate ln(x) to ln(P) and a relevant molecular descriptor of HBDs by the replacement of the “b” parameter. In order to investigate the effect of HBD molecular structure on the CO_2_ solubility, eight separate datasets have been applied with fixed temperature considering Eq. (3):

3 $$\mathrm{ln}\left(x\right)=a\times \mathrm{ln}\left(P\right)+F\left(HBD \,descriptors\right)+c.$$

The CO_2_ solubility values can be predicted only in the fixed temperature (i.e., 293, 303 313, or 323 K) using Eq. (3). In order to take into account the effect of temperature along with the descriptor and ln(P), Eq. (4) has been considered by the replacement of the "c" parameter in Eq. (3) with “ $b\times T$” term. According to the observed trend for the CO_2_ solubility with temperature (see Fig. S3 in the supplementary materials), T was considered as a linear variable in the developed models taking into account the effect of temperature:

4 $$\mathit{ln}\left(x\right)=a\times \mathit{ln}\left(P\right)+b\times T+F\left(HBD\, descriptors\right)+d$$

#### Model development strategy

In the present study, two types of QSPR models have been developed. Equation (3) is applied for the development of the model for the fixed temperature datasets. Equation (4) is applied for the development of the model taking into account the temperature effect on the CO_2_ solubility. Using Eq. (4), the multiple linear regression (MLR) model with three variables (i.e., ln(P), T, and the molecular descriptor of HBDs) was used to derive a predictive and descriptive QSPR model. It is important to note that the suitable descriptor of HBDs should be selected from a set containing various different HBD descriptors (i.e., 444), the ln(P), and T variables. Variable selection for QSPR models can be performed following several approaches^54^. In this study, the Genetic Algorithm (GA) was applied to select the variables of the QSPR model. Further information on the genetic algorithm-multiple linear regression (GA-MLR) can be seen elsewhere^55,56^. It should be noted that the GA-MLR models were built using QSARINS software^57^.

#### Validation of developed models

The estimation capability of all QSPR models should be assessed by implementing internal predictive performance and external predictive performance evaluations. The training set is used for the internal validation, while the test set is used to conduct the external validation. There are several statistical parameters that can be applied to examine the capability of the constructed QSPR model, including the coefficient of determination (R^2^), adjustable coefficient of determination (R^2^_adj_), the standard error (S), the Fisher criterion (F), the Root Mean Square Error (RMSE), Leave One Out Cross-Validated coefficient of determination (Q^2^_LOO-CV_) and the average absolute relative deviation (AARD%). More details on the statistical parameters are provided in the supporting information file (i.e. Table S3 in the supplementary file). In the present study, both internal and external validation methods have been applied. The outcome of such analysis is presented in the following section.

## Results

### The developed QSPR models

Table 2 shows the developed models for unfixed temperature (datasets no. (1) and (2)) and fixed temperature (datasets no. (3)–(10)).

**Table 2:** The obtained QSPR models for fixed and unfixed temperatures datasets after train and test categorization.

No. of dataset	T	HBA to HBD molar ratio	Developed models	Eq. number
1	Unfixed temperatures	1:3	ln(x) = 1.0284(± 0.0806) (ln(P)) − 5.8136(± 0.0939)	(5)
			ln(x) = − 0.0144(± 0.0037) (T) + 1.0382(± 0.0676) (ln(P)) − 1.3674(± 1.1349)	(6)
			ln(x) = 1.0372(± 0.029) (ln(P)) − 0.0171(± 0.0016) (T) + 0.3067 (± 0.0244)(EEig02d) − 1.134(± 0.4879)	(7)
2	Unfixed temperatures	1:4	ln(x) = 1.034(± 0.0873) (ln(P)) − 5.7909(± 0.1016)	(8)
			ln(x) = − 0.0142(± 0.0041) (T) + 1.0408(± 0.076) (ln(P)) − 1.4155(± 1.2787)	(9)
			ln(x) = 1.043(± 0.0318) (ln(P)) − 0.0172(± 0.0017) (T) + 0.3475 (± 0.0268)(EEig02d) − 1.1527(± 0.5351)	(10)
3	293	1:3	ln(x) = 1.0211(± 0.1284) (ln(P)) −5.6132(± 0.1459)	(11)
			ln(x) = 1.036(± 0.0813) (ln(P)) + 0.4228(± 0.1294) (EEig02d) − 6.3254(± 0.2367)	(12)
4	293	1:4	ln(x) = 1.0306(± 0.1254) (ln(P)) −5.5946(± 0.1423)	(13)
			ln(x) = 1.0402(± 0.0679) (ln(P)) + 0.4425(± 0.1074) (EEig02d)-6.3344(± 0.1954)	(14)
5	303	1:3	ln(x) = 1.043(± 0.1452) (ln(P)) − 5.7131(± 0.1694)	(15)
			ln(x) = 1.0339(± 0.0506) (ln(P)) + 0.3113(± 0.0396) (EEig02d) − 6.3119(± 0.0963)	(16)
6	303	1:4	ln(x) = 1.05(± 0.1706) (ln(P)) − 5.6983(± 0.1989)	(17)
			ln(x) = 1.0455(± 0.0644) (ln(P)) + 0.3446(± 0.0481) (EEig02d) − 6.3667(± 0.1198)	(18)
7	313	1:3	ln(x) = 1.0313(± 0.1448) (ln(P)) − 5.8667(± 0.1696)	(19)
			ln(x) = 1.0327(± 0.0529) (ln(P)) + 0.3065(± 0.0412) (EEig02d) − 6.4667(± 0.1017)	(20)
8	313	1:4	ln(x) = 1.0377(± 0.1654) (ln(P)) − 5.842(± 0.1925)	(21)
			ln(x) = 1.0579(± 0.0646) (ln(P)) + 0.3565(± 0.0517) (EEig02d) − 6.5582(± 0.1282)	(22)
9	323	1:3	ln(x) = 1.0546(± 0.1386) (ln(P)) − 6.0674(± 0.1637)	(23)
			ln(x) = 1.0509(± 0.0571) (ln(P)) + 0.2785(± 0.0431) (EEig02d) − 6.6073(± 0.1075)	(24)
10	323	1:4	ln(x) = 1.0422(± 0.1587) (ln(P)) − 6.0222(± 0.1879)	(25)
			ln(x) = 1.0312(± 0.0678) (ln(P)) + 0.3258(± 0.0526) (EEig02d) − 6.6473(± 0.129)	(26)

It was surprising that the same descriptor (i.e. “EEig02d”) appears in all developed models at the fixed and unfixed temperatures. The descriptor “EEig02d” is a molecular descriptor derived from the edge adjacency matrix of a molecule that is weighted by dipole moments. The “EEig02d” descriptor is related to the molar volume of the molecule^58^.

As can be seen in Table 2 and for datasets no. (3)–(10), the best combinations of the ln(P) variable and selected descriptor have been obtained for each fixed temperature (i.e. 293, 303, 313, or 323 K) with their corresponding molar ratio (i.e., 1:3 and 1:4). Besides, the models containing three variables (i.e. ln(P), T, and selected descriptor) have been developed for the unfixed temperature datasets.

It should be mentioned that the developed models (i.e., Eqs. (11)–(26)) for fixed temperature datasets can be applied for the related temperature 293, 303, 313, or 323 K. While the unfixed temperatures models (i.e. Equations (6), (7), (9), (10)) can be used to take into account the effect of temperature on the CO_2_ solubility.

### Validation of the models and statistical evaluation

According to Sarmad et al.^20^, the correlation between ln(x) and ln(P) has been tested for each involved system in any datasets (Please see Table S4 and Fig. S2 in the supplementary file).

In order to evaluate the performance of the developed QSPR models, external validation should be performed. First, data splitting into training and test sets have been created by the Principal component analysis (PCA) method^59^. According to the PCA analysis, for all datasets, the test sets should be chosen in such a way to contain some new structures compared to the train set.

Regarding the datasets no. (3) and no. (4), all related data of one structure of HBD (i.e., Diethylene glycol) was set aside in the test set due to the shortage of the structural variations. Unlike the datasets no. (3) and no. (4), it should be added that all related data of two structures of HBD (i.e. Furfuryl alcohol and Diethylene glycol) were considered as the test set for the other datasets (i.e. (5)–(10)). It should be added that all available datapoints in two remaining datasets (i.e. no. (11) and (12)) were considered only for further external validation. Then, it was tried to choose the most appropriate molecular descriptor of HBD as Eq. (3) for fixed temperature datasets and Eq. (4) for unfixed temperature datasets. As shown in Table 2, the obtained models with one or two variables, and one, two, or three variables have been presented for fixed and unfixed temperature datasets, respectively. The appeared descriptor in each developed QSPR model was the same (i.e. “EEig02d”). The values of statistical parameters for either fixed or unfixed models are given in Table 3 for the train and test sets.

**Table 3:** Statistical parameters of the obtained models for each dataset in logarithm and non-logarithm scale separately.

Model base	No. of dataset	Eq. nos	Name of set	R^2^	R^2^adj	S	F	RMSE	Q^2^loo	AARD
Ln-based	1	(5)	Train	0.8198	0.8185	0.2897	636.84	0.2877	0.8144	5.0039
			Test	0.9309				0.1722		2.9413
		(6)	Train	0.8743	0.8725	0.2428	483.42	0.2402	0.8688	4.2937
			Test	0.9863				0.0782		1.2110
		(7)	Train	0.977	0.9765	0.1043	1951.53	0.1028	0.9757	1.8383
			Test	0.976				0.1161		1.927
	2	(8)	Train	0.7965	0.7951	0.3144	548.08	0.3122	0.7906	5.3764
			Test	0.9166				0.192		3.2552
		(9)	Train	0.8471	0.8449	0.2735	385.096	0.2706	0.8406	4.6347
			Test	0.9645				0.1269		2.3089
		(10)	Train	0.9735	0.9729	0.1144	1687.46	0.1127	0.972	2.0557
			Test	0.9468				0.19		3.1058
	3	(11)	Train	0.9012	0.8978	0.2108	264.5	0.2038	0.8876	4.0477
			Test	0.999				0.0568		0.9224
		(12)	Train	0.962	0.9593	0.133	354.34	0.1264	0.9551	2.5226
			Test	0.999				0.0776		1.5367
	4	(13)	Train	0.9069	0.9037	0.2045	282.64	0.1977	0.8937	3.7408
			Test	1				0.0394		0.8076
		(14)	Train	0.9737	0.9719	0.1106	518.95	0.1051	0.9691	1.895
			Test	1				0.0351		0.5167
	5	(15)	Train	0.8586	0.8546	0.2621	212.6	0.255	0.8399	4.787
			Test	0.9955				0.055		1.0481
		(16)	Train	0.9834	0.9824	0.0912	1005.91	0.0874	0.9808	1.6286
			Test	0.9888				0.1047		1.707
	6	(17)	Train	0.8169	0.8116	0.2938	156.1	0.2857	0.7923	5.3186
			Test	0.9643				0.1204		2.6312
		(18)	Train	0.9747	0.9732	0.1108	654.8	0.1062	0.9707	1.9308
			Test	0.9458				0.1858		3.3345
	7	(19)	Train	0.8566	0.8525	0.26	209.12	0.2528	0.8378	4.4939
			Test	0.9941				0.0527		0.8675
		(20)	Train	0.9814	0.9803	0.0949	898.72	0.091	0.9789	1.6453
			Test	0.9868				0.108		1.7963
	8	(21)	Train	0.8226	0.8175	0.3052	162.31	0.2969	0.7987	4.9231
			Test	0.9747				0.1076		2.0045
		(22)	Train	0.9738	0.9723	0.119	632.42	0.114	0.9696	2.18
			Test	0.9599				0.1956		3.0558
	9	(23)	Train	0.8721	0.8685	0.2411	238.7	0.2345	0.8564	3.9275
			Test	0.9715				0.1123		1.7689
		(24)	Train	0.9789	0.9777	0.0993	789.49	0.0952	0.9756	1.644
			Test	0.9589				0.1433		2.4663
	10	(25)	Train	0.8355	0.8308	0.2841	177.82	0.2763	0.8159	4.485
			Test	0.9408				0.1659		3.0796
		(26)	Train	0.9709	0.9692	0.1212	567.38	0.1162	0.9664	2.1414
			Test	0.9215				0.2258		3.8977
Non Ln-based	1	(7)	Train	0.9571	0.9562	0.0013	1048.75	0.0013	–	8.4735
			Test	0.9699				0.0011	–	8.718
	2	(10)	Train	0.9553	0.9543	0.0014	856.5	0.0014	–	9.6184
			Test	0.9288				0.002	–	13.077
	3	(12)	Train	0.9377	0.9333	0.0017	198.06	0.0016	–	11.4934
			Test	0.9989				0.0008	–	7.5609
	4	(14)	Train	0.9569	0.9538	0.0015	291.87	0.0014	–	8.7299
			Test	0.9999				0.0002	–	2.6093
	5	(16)	Train	0.9696	0.9678	0.0013	558.27	0.0012	–	7.333
			Test	0.9821				0.0011	–	7.3524
	6	(18)	Train	0.9568	0.9543	0.0017	392.61	0.0016	–	8.5353
			Test	0.9065				0.0025	–	13.2433
	7	(20)	Train	0.9686	0.9668	0.0011	540.28	0.0011	–	7.5851
			Test	0.9805				0.0011	–	7.9223
	8	(22)	Train	0.9588	0.9564	0.0014	408.38	0.0013	–	10.2979
			Test	0.9504				0.0018	–	12.7557
	9	(24)	Train	0.9657	0.9637	0.0009	496	0.0009	–	7.9132
			Test	0.9558				0.001	–	11.5962
	10	(26)	Train	0.9637	0.9616	0.0011	468.38	0.001	–	10.6182
			Test	0.9028				0.0019	–	17.2659

According to Table 3, the prediction capability of the developed models with two-variables (i.e. ln(P) and “EEig02d”), which considered the effect of HBD structures (i.e. Eqs. (12), (14), (16), (18), (20), (22), (24), and (26)), is superior compared to one-variable (i.e. ln(P)) models (i.e. Eqs. (11), (13), (15), (17), (19), (21), (23), and (25)) considering the fixed temperature datasets. Moreover, the one-variable (i.e. ln(P)) and two-variables (i.e. ln(P) and T) models (i.e. Eqs. (5), (6), (8), (9)) are not appropriate for unfixed temperature datasets because they cannot take into account the effect of HBD structure on the CO_2_ solubility. Then, it is essential to add a molecular variable along with other variables to distinguish the effect of different structures of the HBDs on the CO_2_ solubility (Eqs. (7) and (10)). It can be concluded that considering the effect of HBD structure using the “EEig02d” descriptor improved the estimation of CO_2_ solubility significantly. It should be mentioned that the values of statistical parameters in the non-logarithm scale have been reported along with the logarithm scale in Table 3.

The experimental versus the predicted values of CO_2_ solubility are shown in Figs. 1 and 2 for dataset no. (1) with variable temperature and dataset no. (5) with fixed temperature, respectively. These figures for other datasets can be found in the supporting information file (Figs. S4a–S13a).

**Figure 1:**
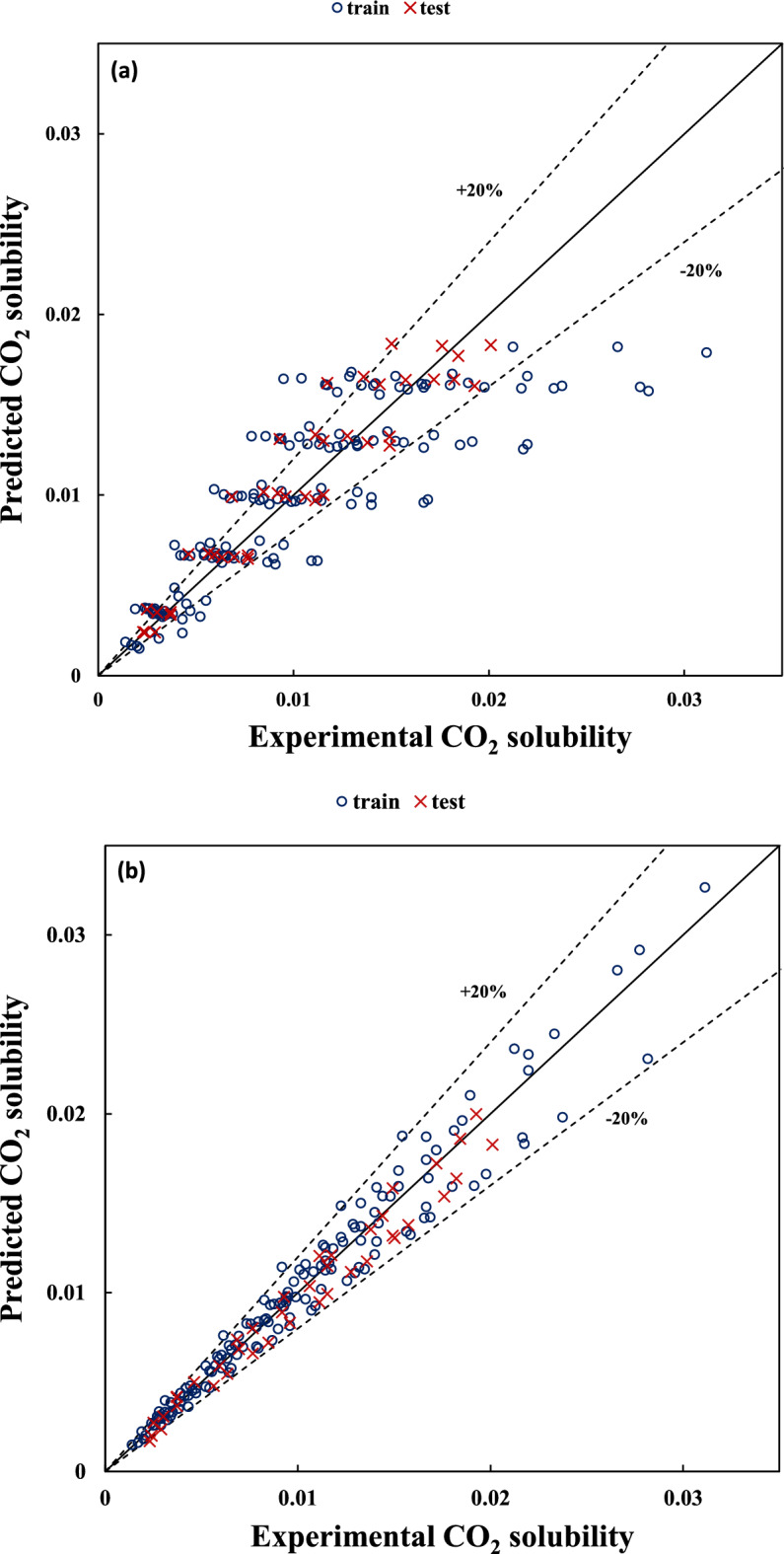
Predicted versus experimental values of CO_2_ solubility for unfixed temperatures dataset (i.e. dataset no. (1)) using (**a**) Eq. (5) and (**b**) Eq. (7).

**Figure 2:**
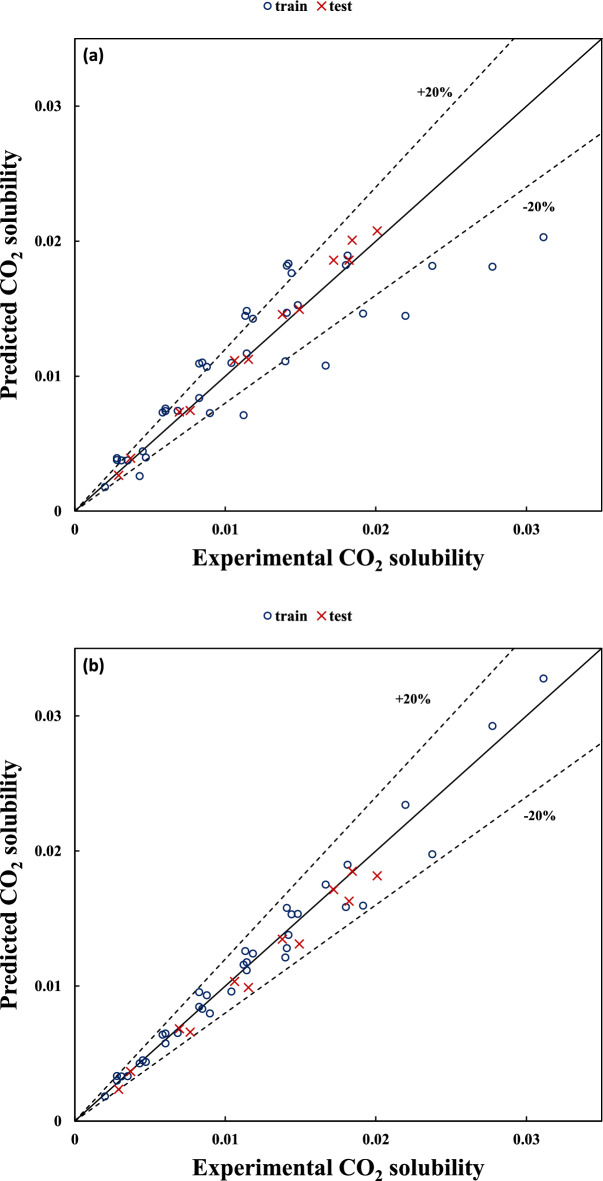
Predicted versus experimental values of CO_2_ solubility for a fixed temperature dataset (i.e. dataset no. (5)) using (**a**) Eq. (15) and (**b**) Eq. (16).

As can be seen in Figs. 1a and 2a, the prediction capability of models using Eqs. (5) and (15) is not acceptable because these models only consider the effect of pressure on the CO_2_ solubility. However, according to Figs. 1b and 2b, taking into account the HBD structural effect in Eqs. (7) and (16) lead to a considerable enhancement in the estimation of CO_2_ solubility for both train and test sets.

**Figure 3:**
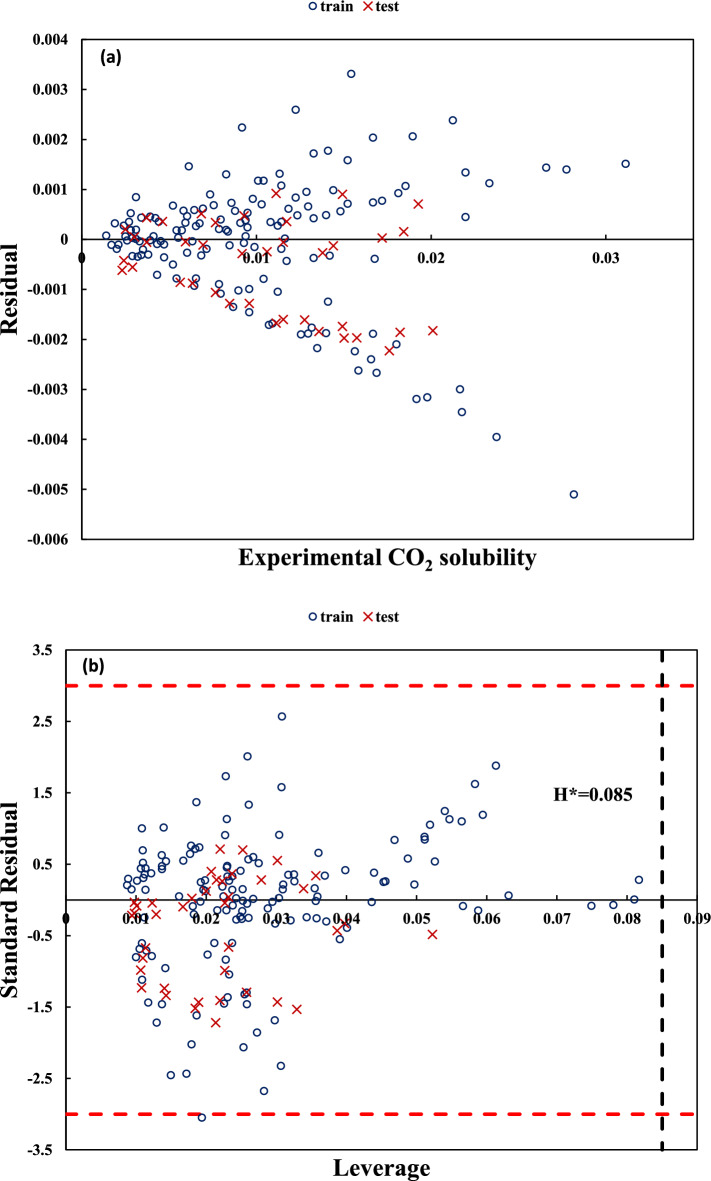
Residuals versus experimental values of CO_2_ solubility (**a**) and Standard residuals versus leverage (**b**) for unfixed temperatures dataset (dataset no. (1)) using Eq. (7).

**Figure 4:**
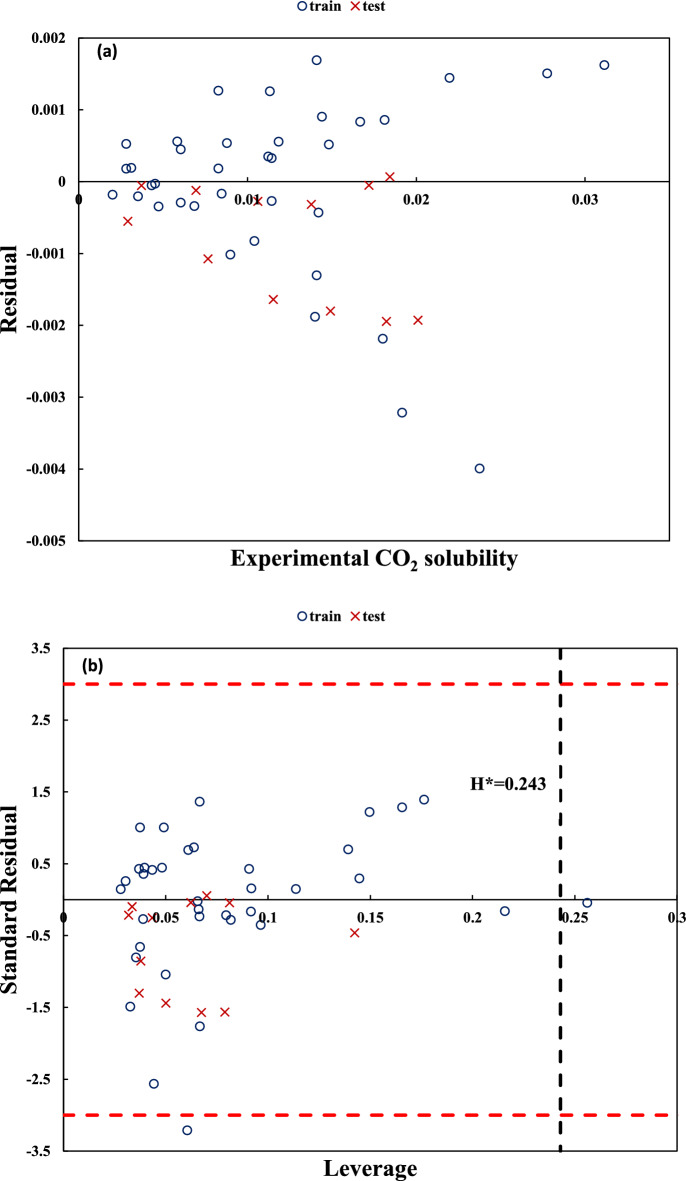
Residuals versus experimental values of CO_2_ solubility (**a**) and Standard residuals versus leverage (**b**) for fixed temperature dataset (dataset no. (5)) using Eq. (16).

Figures 3a and 4a show the experimental versus the residual values of CO_2_ solubility for dataset no. (1) using Eq. (7) and dataset no. (5) using Eq. (16), respectively. As can be observed, a normal distribution of the residual values for train and test data is achieved. Figures 3b and 4b show the standard error versus leverage values (i.e. William’s plot) for dataset no. (1) with variable temperature and dataset no. (5) with fixed temperature. As can be observed, there is no outlier data for these datasets. These figures can be used to identify the applicability domain of the constructed models. Additional figures corresponding to the remaining datasets are available in the supplementary file (part b and c of Figs. S4–S13a).

According to the developed models, the “EEig02d” descriptor is the appropriate structural variable for the prediction of solubility of CO_2_. It is clear that the “EEig02d” descriptor appeared in all models, so it can be concluded that it was not selected randomly. The values of the predicted CO_2_ solubility by the QSPR models mentioned in Table 2 for each data point of all datasets are available in the supporting Excel file. Table 3 presents the outcome of the statistical examination of the constructed models. As can be observed in Table 3, the models including the EEig02d descriptor, showed the best statistical parameters in both logarithmic and non-logarithmic scales considering both internal and external validations.

**Figure 5:**
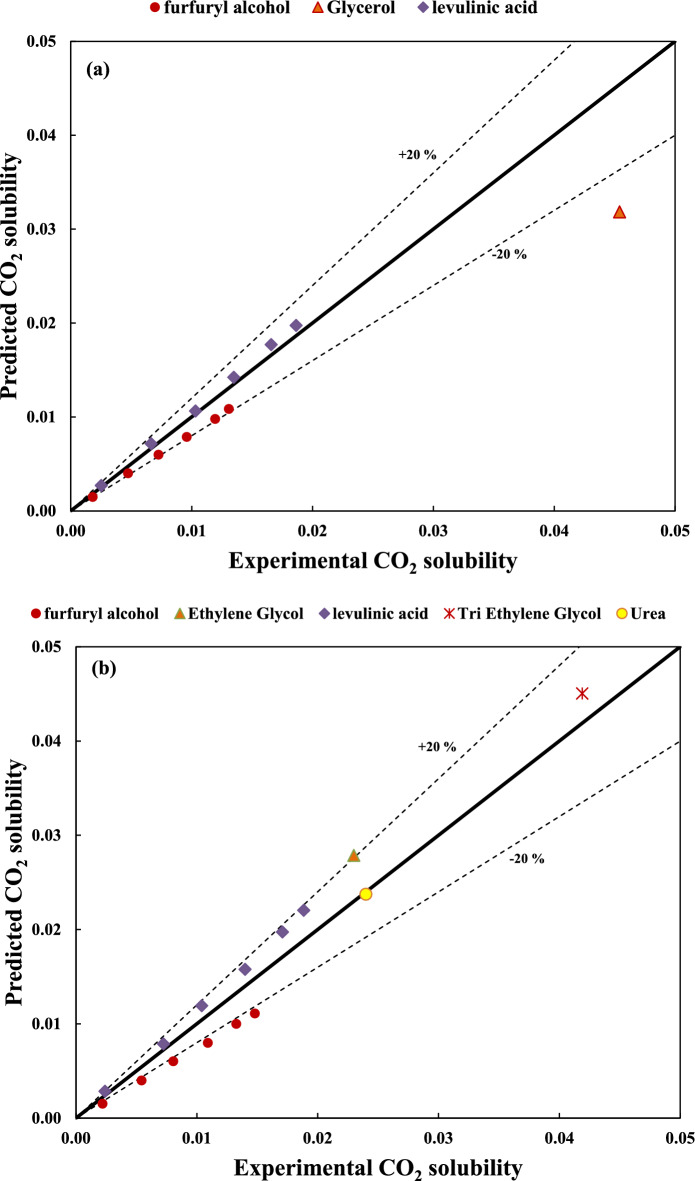
Predicted versus experimental values of CO_2_ solubility for (**a**) dataset no. (11)) using Eq. (7) and (**b**) dataset no. (12) Eq. (10).

In order to investigate the applicability of the unfixed temperature models in new temperatures and pressures, datasets no. (11) and (12) were used. In other words, these datasets contain some new HBDs (i.e. Glycerol in dataset no (11) and Urea and Ethylene glycol in dataset no. (12)). Moreover, both datasets have some new temperatures (i.e., 298 and 333 K) and pressures (i.e. 10 bar) which were different comparing the datasets no. (1) and (2) applied for the model development. According to Fig. S14 in the supplementary word file, all datapoints in these two new datasets were in the domine of applicability, Therefore, Eq. (7) and (10) for dataset no. (11) and (12) can be applied, respectively. Figure 5 shows the experimental versus the predicted values of CO_2_ solubility for dataset no. (11) using Eq. (7) and dataset no. (12) using Eq. (10), respectively. Surprisingly, the proposed models showed very good capability for the prediction of solubility at low pressure (i.e. low solubility). At high pressure (i.e., high solubility) the prediction of solubility shows an acceptable deviation, which confirms the robustness and applicability of the models at different temperatures and pressures even for new structures.

## Discussion

**Figure 6:**
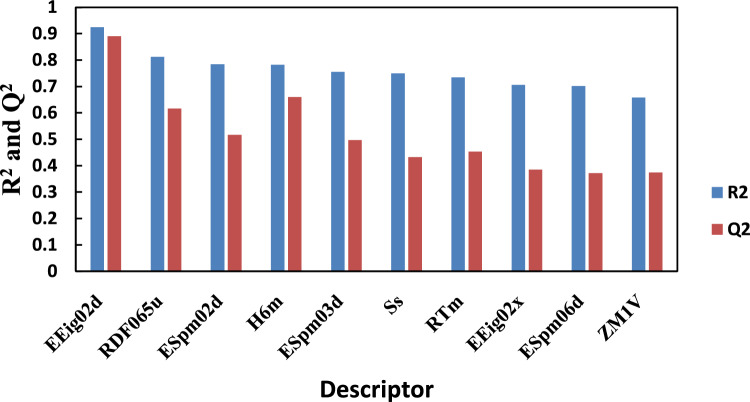
R^2^ and Q^2^ for sub-dataset with P = 5 bar, T = 313 K and molar ratio 1:4.

It should be proven that the selected descriptor has the best performance for the prediction of the CO_2_ solubility. In this regard, some sub-datasets have been selected randomly from the datasets no. (1) and (2) in such a way that in each sub-dataset temperature, pressure and molar ratio was almost constant and only the structure of HBDs was variable. Then, some models with only one variable (i.e., structural descriptor) have been developed and compared statistically. For instance, Fig. 6 shows the values of R^2^ and Q^2^ for one of these sub-datasets consisting data with pressure approximately 5 bar, temperature of 313 K and HBA to HBD molar ratio of 1:4. The figures corresponding to other sub-datasets are shown in the supplementary word file.

As it is clear from Fig. 6 and Fig. S15, there are several models such that their statistical parameters satisfy the Golbraikh criterion (R^2^ > 0.6 and Q^2^ > 0.5)^60^. The values of descriptors with acceptable statistical parameters are indicated in Table 4. The values of some descriptors (i.e. H6m and RDF065u) are zero for several HBDs. It means that these descriptors are not appropriate for the model development because these descriptors cannot distinguish between some structures. Apart from this point, it is obvious that it is better to choose a descriptor that is not only repeated in all of the sub-datasets, but have acceptable statistical parameters. Therefore, it is confirmed that the selected descriptor (i.e., EEig02d) is an appropriate molecular descriptor in the developed models.

**Table 4:** Values of descriptors for each involved HBDs.

HBD	H6m	RDF065u	RTm	ESpm02d	EEig02d	Ss	ZM1V
1,2-Propanediol	0	0	1.953	2.349	1.054	16.83	64
1,4-Butanediol	0	0	2.032	2.349	1.519	18	66
2,3-Butanediol	0	0	2.289	2.673	1.519	18.67	70
Diethylene glycol	0.003	0.052	1.879	2.636	1.713	21.5	102
Furfuryl alcohol	0	0.978	2.86	3.228	1.983	22.83	130
Guaiacol	0	0	2.187	2.876	1.521	17.67	86
Levulinic acid	0.004	1.207	2.335	3.065	2.242	28	146
Phenol	0	0.157	2.377	2.902	1.547	18.67	108
Triethylene glycol	0.01	1.861	3.469	3.423	3.515	28.33	138

After model development, the molecular descriptor that appeared in the QSPR models (i.e. “EEig02d”) should be interpreted to explain why it is related to the CO_2_ solubility in DESs. The “EEig02d” descriptor, developed by Estrada et al.^58,61^, corresponds to the second eigenvalue of the edge adjacency matrix of the molecule, which is weighted by dipole moments of atoms. The edge adjacency matrix is ​​obtained through a hydrogen-depleted molecular graph, a graph whose nodes are related to the atoms of the molecule and edges are related to the chemical bonds. The molecular graphs are converted into mathematical expression like matrices to correlate the structure and properties quantitatively. The edge-adjacency matrix (EA(G)) of a graph G is defined as follows^62^:

27 $${(\mathrm{EA})}_{\mathrm{ij}}=\left\{\begin{array}{l}1\, \quad if\, edges\, {\mathrm{e}}_{\mathrm{i}}\,and\, {\mathrm{e}}_{\mathrm{j}}\, are\, adjacent\\ 0\, \quad otherwise\end{array}\right.$$

28 $${(\mathrm{EA})}_{\mathrm{ii}}=0$$

For the adjacency matrix of a weighted graph, Eq. (27) should be modified as Ref.^62^:

29 $${(EA)}_{ij}=\left\{\begin{array}{l}K\,\quad if\, edges\, {\mathrm{e}}_{\mathrm{i}}\, and\, {\mathrm{e}}_{\mathrm{j}}\, are\, adjacent\, and \,{\mathrm{e}}_{\mathrm{j}}\, is\, K-weighted\\ 0\,\quad otherwise\end{array}\right.,$$

where e_i_ and e_j_ are the chemical bonds, and K is the weights of edges.

Table 5 shows the values of EEig02d along with the molar volume and the molecular structure of all HBDs involved in the datasets. It should be mentioned that the EEig02d descriptor can be related to the molar volume of the molecule^58^.

**Table 5:** The values of the EEig02d and molar volume of the different HBDs involved in the datasets.

HBD	EEig02d	V (cm^3^/mole)	Structure
1,2-Propanediol	1.054	72.93	
1,4-Butanediol	1.519	88.81	
2,3-Butanediol	1.519	89.69	
Diethylene glycol	1.713	95.05	
Furfuryl alcohol	1.547	86.37	
Guaiacol	1.983	109.53	
Levulinic acid	3.515	102.45	
Phenol	1.521	87.96	
Triethylene glycol	2.242	133.63	

It is plausible that the values of the EEig02d increase by increasing the length of the alkyl chain of HBD. For example, the value of EEig02d for 1,2-Propanediol with three carbons in its alkyl chain and 1,4-Butanediol and 2,3-Butanediol with four carbons in their alkyl chains are 1.054 and 1.519, respectively. It is also observed that the presence of the ether group also increases the value of the EEig02d descriptor. In this regard, the values of the EEig02d for guaiacol are higher compared to phenol (1.983 versus 1.521), due to the presence of the ether group in guaiacol structure. It should be noted that increasing the length of the alkyl chain results in an increment in the molecular free volume in the DESs. Also, the presence of ether groups increases the flexibility of the alkyl chain and thus leads to an increase in the free volume, and consequently enhances the solubility of CO_2_ in DES because of the physical nature of absorption (i.e. free volume mechanism)^16,20^.

Moreover, according to Li et al.^24^, the increment of pressure and temperature have a positive and a negative effect on the CO_2_ solubility, respectively. These findings are consistent with the developed models indicated in Table 2 since EEig02d and the pressure have appeared with a positive sign, and the temperature has appeared with a negative sign. The enhancement in CO_2_ solubility by increasing the length of the alkyl chain group was also demonstrated by experimental works.

## Conclusion

In the current study, QSPR approach was employed to develop linear models for predicting the CO_2_ solubility in the DESs. The main aim was to investigate the effect of the structure of HBDs on the solubility of CO_2_ in the ChCl-based DESs. The main findings are as follows:

- It was noteworthy that the same descriptor (i.e. EEig02d) along with ln(P) appeared in all developed models, independent of the effect of temperature. It was found that the EEig02d descriptor is related to the molar volume and dipole moment of a molecule. Examination of the models indicated that the solubility increases with increasing the values of the EEig02d descriptor because there is a direct relationship between physical absorption and the free volume of the molecule.
- Two general models in HBA to HBD molar ratios equal to 1:3 and 1:4 were constructed by the combination of ln(P), T, and EEig02d as the structural descriptor variable to predict the CO_2_ solubility in ChCl-based DESs at any desired temperature. These models were examined by further external validation using two additional datasets containing new HBD structures.
- This study provided reliable and simple QSPR models for predicting the CO_2_ solubility in ChCl-based DESs, which can be applied in the preliminary screening of the DESs in the PCC processes.

## Supplementary Information

Supplementary Information 1.

Supplementary Information 2.

## Data Availability

It should be justified that “All data generated or analysed during this study are included in this published article [and its supplementary information files]”.
