# Supplementary material for: New molecular structure based models for estimation of the CO2 solubility in different choline chloride-based deep eutectic solvents (DESs)
Source: Sci Rep. 2023 May 25;13:8495. doi: 10.1038/s41598-023-35747-8 (PMC10213022; doi:10.1038/s41598-023-35747-8)
Supplement: Supplementary file 1 — Supplementary Information 1. [file 41598_2023_35747_MOESM1_ESM.docx]

# Supplementary materials

# For

**New Molecular structure based models for estimation of the CO_2_ solubility in different choline chloride-based deep eutectic solvents (DESs)**

# Farnoosh Dehkordi, Mohammad Amin Sobati*, Ali Ebrahimpoor Gorji

School of Chemical Engineering, Iran University of Science and Technology (IUST), Tehran, Iran

*Corresponding author: Phone: +98 (21) 77240496, Fax: +98 (21) 77240495

E-mail:[sobati@iust.ac.ir](mailto:sobati@iust.ac.ir)

**Supporting information**

Table S 1: Available QSPR models in the literature for the prediction of different properties of DESs

| **Reference** | **Property of DES** | **Statistical parameter** | |
| --- | --- | --- | --- |
| Khajeh et al. [1] | melting points | R^2^= 0.795 | |
| Khajeh et al. [1] | freezing points | R^2^= 0.764 | |
| Lemaoui et al. [2] | Viscosity | R^2^=0.9874 | |
| Lemaoui et al. [2] | Density | R^2^=0.9839 | |
| Benguerba et al. [3] | Viscosity | ANN | R^2^=0.9863 |
|  |  | MLR | R^2^=0.9305 |
| Lemaoui et al. [4] | Electrical conductivity | R^2^=0.985 | |
| Halder et al. [5] | Surface Tension | MLR | R^2^=0.916 |
|  |  | SVM | R^2^=0.874 |
| Mjalli et al. [6] | Surface Tension | R^2^=0.9952 | |
| Kurnia et al. [7] | Viscosity | R^2^=0.9734 | |
| Halder et al. [8] | Viscosity | R^2^=0.877 | |

Table S 2: Applied different thermodynamic methods for the modeling of the solubility of CO2 in different DESs.

| **Reference** | **PR** | **PC-SAFT** | **CPA** | **NRTL** | **COSMO-RS** | **RK** | **Monte Carlo** | **DFT** | **MD** | **QSPR** |
| --- | --- | --- | --- | --- | --- | --- | --- | --- | --- | --- |
| Alioui et al. [9] |  |  |  |  | √ |  |  |  |  |  |
| Sarmad et al. [10] |  |  |  |  |  | √ |  |  |  |  |
| Haider et al. [11] | √ |  |  | √ |  |  |  |  |  |  |
| Salehi et al. [12] |  |  |  |  |  |  | √ |  |  |  |
| Haider et al. [13] | √ |  |  |  |  |  |  |  |  |  |
| Mirza et al. [14] | √ |  |  |  |  |  |  |  |  |  |
| Haghbakhsh et al. [15] |  |  | √ |  |  |  |  |  |  |  |
| Altamash et al. [16] |  |  |  |  |  |  |  |  | √ |  |
| Dietz et al. [17] |  | √ |  |  |  |  |  |  |  |  |
| Kamgar et al. [18] |  |  |  | √ | √ |  |  |  |  |  |
| Zubeir et al. [19] |  | √ |  |  |  |  |  |  |  |  |
| Ullah et al. [20] |  |  |  |  |  |  |  | √ | √ |  |
| Garcia et al. [21] |  |  |  |  |  |  |  | √ |  |  |
| Garcia et al. [22] |  |  |  |  |  |  |  | √ | √ |  |
| Ali et al. [23] | √ |  |  |  |  |  |  |  |  |  |
| Xie et al. [24] |  |  |  | √ |  | √ |  |  |  |  |
| Liu et al. [25] |  |  |  |  | √ |  |  |  |  |  |
| Wang et al. [26] |  |  |  |  |  |  |  |  |  | √ |
| Kumar et al. [27] |  |  |  |  |  |  |  |  |  | √ |
| Halder et al. [8] |  |  |  |  |  |  |  |  |  | √ |
| Present study |  |  |  |  |  |  |  |  |  | √ |

Table S 3: Applied statistical parameters in this study

| Introduced parameters | | | Introduced parameters equations^*^ | Eqs. No | |
| --- | --- | --- | --- | --- | --- |
| Coefficient of determination | | $R^{2}=1-\frac{\sum_{i=1}^{n} (y_{i}^{\exp}-y_{i}^{\mathrm{cal}})^{2}}{\sum_{i=1}^{n} (y_{i}^{\exp}-\overline{y^{\exp}})^{2}}$ | | | (S1) |
| Adjustable coefficient of determination | $R_{\mathrm{adj}}^{2}=1-\frac{(1-R^{2})(n-1)}{(n-P-1)}$ | | | | (S2) |
| Leave-one-out cross-validated coefficient of determination | $Q_{loo-cv}^{2}=1-\frac{\sum_{i=1}^{n} (y_{i}^{\exp}-y_{i}^{\mathrm{cal}})^{2}}{\sum_{i=1}^{n} (y_{i}^{\exp}-\overline{y^{\exp}})^{2}}$ | | | | (S3) |
| Fisher function | $F=\frac{{\sum_{i=1}^{n} (y_{i}^{\mathrm{cal}}-\overline{y^{\exp}})^{2}}/P}{{\sum_{i=1}^{n} (y_{i}^{\exp}-y_{i}^{\mathrm{cal}})^{2}}/{(n-P-1)}}$ | | | | (S4) |
| Average Absolute Relative Deviation (AARD %) | $\mathrm{AARD}\left( \% \right)=\frac{1}{n}\sum_{i=1}^{n} \left\vert\frac{y_{i}^{\exp}-y_{i}^{\mathrm{cal}}}{y_{i}^{\exp}} \right\vert\times100$ | | | | (S5) |
| Standard error (S) | $S=\sqrt{\frac{\sum_{i=1}^{n} (y_{i}^{\mathrm{cal}}-y_{i}^{\exp})^{2}}{n-P-1}}$ | | | | (S6) |
| Root Mean Square Error (RMSE) | $\mathrm{RMSE}_{\mathrm{CV}}=\sqrt{\frac{1}{n}\sum_{i=1}^{n} (y_{i}^{\exp}-y_{i}^{\mathrm{cal}})^{2}}$ | | | | (S7) |
| maximum Leverage | $H^{*}=3(P+1)/n$ | | | | (S8) |

^*^ Where $y_{i}^{\exp}$, $y_{i}^{\mathrm{cal}}$, $\bar{y}_{i}^{\exp}$ n, p represent experimental value of ln(x), predicted value of ln(x), average experimental value of ln(x), number of data in the train or test sets, and number of the variables in the model, respectively.

Table S 4: The variation ranges of ln(P), and ln(x) and the correlation between ln(x) and ln(P) for each involved system in any datasets.

| **T** | **Ratio** | **HBD** | **ln(P)-range** | **ln(X)-range** | **no. of data** | **R^2^** | **ref.** |
| --- | --- | --- | --- | --- | --- | --- | --- |
| 293 | 1:3 | 1,2-Propanediol | (0.0816)-(1.638) | (-5.7138)-(-4.0864) | 5 | 0.9999 | [28] |
|  |  | 1,4-Butanediol | (0.1035)-(1.6287) | (-5.684)-(-4.0923) | 5 | 0.9994 | [28] |
|  |  | 2,3-Butanediol | (0.1337)-(1.6318) | (-5.7764)-(-4.1734) | 5 | 0.9999 | [28] |
|  |  | Diethylene glycol | (0.1204)-(1.6349) | (-5.5994)-(-3.9477) | 5 | 0.999 | [29] |
|  |  | Guaiacol | (-0.6636)-(1.6666) | (-6.1658)-(-3.8167) | 6 | 0.9997 | [30] |
|  |  | Phenol | (0.0431)-(1.6263) | (-5.4491)-(-3.8304) | 5 | 0.9995 | [29] |
|  |  | Triethylene glycol | (0.0889)-(1.6174) | (-5.2591)-(-3.5684) | 5 | 0.9996 | [29] |
|  |  | Regardless of the effect of changing HBD structures | (-0.6636)-(1.6666) | (-6.1658)-(-3.5684) | 36 | 0.9115 | [28-30] |
|  |  |  |  |  |  |  |  |
| 293 | 1:4 | 1,2-Propanediol | (0.0431)-(1.6124) | (-5.7764)-(-4.0864) | 5 | 0.9992 | [28] |
|  |  | 1,4-Butanediol | (0.0908)-(1.6359) | (-5.7764)-(-4.1605) | 5 | 1 | [28] |
|  |  | 2,3-Butanediol | (0.0686)-(1.6263) | (-5.5728)-(-3.9528) | 5 | 0.9984 | [28] |
|  |  | Diethylene glycol | (0.0989)-(1.6269) | (-5.5468)-(-3.868) | 5 | 1 | [29] |
|  |  | Guaiacol | (-0.652)-(1.6601) | (-6.1193)-(-3.7898) | 6 | 1 | [30] |
|  |  | Phenol | (0.0788)-(1.6277) | (-5.4491)-(-3.8258) | 5 | 0.9991 | [29] |
|  |  | Triethylene glycol | (0.174)-(1.6361) | (-5.116)-(-3.5474) | 5 | 0.9997 | [29] |
|  |  | Regardless of the effect of changing HBD structures | (-0.652)-(1.6601) | (-6.1193)-(-3.5474) | 36 | 0.9179 | [28-30] |
|  |  |  |  |  |  |  |  |
| 303 | 1:3 | 1,2-Propanediol | (0.1613)-(1.6429) | (-5.8781)-(-4.2545) | 5 | 0.998 | [28] |
|  |  | 1,4-Butanediol | (0.1231)-(1.6044) | (-5.7764)-(-4.2405) | 5 | 0.9998 | [28] |
|  |  | 2,3-Butanediol | (0.131)-(1.6359) | (-5.8781)-(-4.2616) | 5 | 0.9983 | [28] |
|  |  | Diethylene glycol | (0.163)-(1.7302) | (-5.5994)-(-3.9954) | 6 | 0.9997 | [29, 31] |
|  |  | Furfuryl alcohol | (-0.212)-(1.7627) | (-5.843)-(-3.907) | 6 | 0.9999 | [32] |
|  |  | Guaiacol | (-0.5924)-(1.6737) | (-6.2146)-(-4.0118) | 6 | 0.9997 | [30] |
|  |  | Levulinic acid | (-0.2307)-(1.7405) | (-5.4491)-(-3.4705) | 6 | 0.9998 | [32] |
|  |  | Phenol | (0.1231)-(1.6374) | (-5.655)-(-4.0174) | 5 | 0.9993 | [29] |
|  |  | Triethylene glycol | (0.1765)-(1.6345) | (-5.3602)-(-3.7381) | 5 | 0.9999 | [29] |
|  |  | Regardless of the effect of changing HBD structures | (-0.5924)-(1.7627) | (-6.2146)-(-3.4705) | 49 | 0.8868 | [28-32] |
|  |  |  |  |  |  |  |  |
| 303 | 1:4 | 1,2-Propanediol | (0.1587)-(1.6438) | (-5.9145)-(-4.3351) | 5 | 0.9985 | [28] |
|  |  | 1,4-Butanediol | (0.0564)-(1.6166) | (-5.9145)-(-4.2904) | 5 | 0.999 | [28] |
|  |  | 2,3-Butanediol | (0.1621)-(1.6316) | (-5.655)-(-4.0745) | 5 | 0.9999 | [28] |
|  |  | Diethylene glycol | (0.1467)-(1.6567) | (-5.684)-(-4.0628) | 6 | 0.9999 | [29, 31] |
|  |  | Furfuryl alcohol | (-0.1924)-(1.7604) | (-5.7446)-(-3.7593) | 6 | 0.9998 | [32] |
|  |  | Guaiacol | (0.003)-(1.6973) | (-5.655)-(-3.9221) | 6 | 0.9999 | [30] |
|  |  | Levulinic acid | (-0.3216)-(1.749) | (-5.5468)-(-3.4234) | 6 | 1 | [32] |
|  |  | Phenol | (0.1398)-(1.655) | (-5.655)-(-4.0456) | 5 | 0.9994 | [29] |
|  |  | Triethylene glycol | (0.0889)-(1.6351) | (-5.3185)-(-3.677) | 5 | 0.9998 | [29] |
|  |  | Regardless of the effect of changing HBD structures | (-0.3216)-(1.7604) | (-5.9145)-(-3.4234) | 49 | 0.8511 | [28-32] |
|  |  |  |  |  |  |  |  |
| 313 | 1:3 | 1,2-Propanediol | (0.2263)-(1.6398) | (-6.0323)-(-4.4568) | 5 | 0.9999 | [28] |
|  |  | 1,4-Butanediol | (0.1939)-(1.614) | (-5.8781)-(-4.4063) | 5 | 0.9979 | [28] |
|  |  | 2,3-Butanediol | (0.1765)-(1.6654) | (-5.843)-(-4.3505) | 5 | 1 | [28] |
|  |  | Diethylene glycol | (0.1561)-(1.6404) | (-5.8091)-(-4.2405) | 5 | 0.9995 | [29] |
|  |  | Furfuryl alcohol | (-0.2132)-(1.7608) | (-6.0323)-(-4.0399) | 6 | 0.9999 | [32] |
|  |  | Guaiacol | (-0.5534)-(1.6666) | (-6.3771)-(-4.1865) | 6 | 0.9996 | [30] |
|  |  | Levulinic acid | (-0.3595)-(1.7575) | (-5.7764)-(-3.6268) | 6 | 0.9999 | [32] |
|  |  | Phenol | (0.1484)-(1.6229) | (-5.7446)-(-4.1477) | 5 | 0.9999 | [29] |
|  |  | Triethylene glycol | (0.131)-(1.6304) | (-5.5728)-(-3.9221) | 5 | 0.999 | [29] |
|  |  | Regardless of the effect of changing HBD structures | (-0.5534)-(1.7608) | (-6.3771)-(-3.6268) | 48 | 0.8851 | [28-30, 32] |
|  |  |  |  |  |  |  |  |
| 313 | 1:4 | 1,2-Propanediol | (0.1956)-(1.6594) | (-6.1658)-(-4.5375) | 5 | 0.9983 | [28] |
|  |  | 1,4-Butanediol | (0.1781)-(1.6237) | (-5.9522)-(-4.4228) | 5 | 0.9996 | [28] |
|  |  | 2,3-Butanediol | (0.1035)-(1.6312) | (-5.8091)-(-4.2199) | 5 | 0.9995 | [28] |
|  |  | Diethylene glycol | (0.1527)-(1.6452) | (-5.8091)-(-4.2064) | 5 | 0.9998 | [29] |
|  |  | Furfuryl alcohol | (-0.3524)-(1.7384) | (-5.9915)-(-3.9846) | 6 | 0.9998 | [32] |
|  |  | Guaiacol | (-0.6274)-(1.6918) | (-6.5713)-(-4.0864) | 6 | 0.9983 | [30] |
|  |  | Levulinic acid | (-0.5108)-(1.7332) | (-5.843)-(-3.572) | 6 | 0.9999 | [32] |
|  |  | Phenol | (0.1579)-(1.6184) | (-5.7446)-(-4.1352) | 5 | 0.9998 | [29] |
|  |  | Triethylene glycol | (0.179)-(1.6467) | (-5.4727)-(-3.8825) | 5 | 0.9986 | [29] |
|  |  | Regardless of the effect of changing HBD structures | (-0.6274)-(1.7384) | (-6.5713)-(-3.572) | 48 | 0.8527 | [28-30, 32] |
|  |  |  |  |  |  |  |  |
| 323 | 1:3 | 1,2-Propanediol | (0.207)-(1.6577) | (-6.2659)-(-4.6565) | 5 | 0.9992 | [28] |
|  |  | 1,4-Butanediol | (0.2054)-(1.6599) | (-5.9915)-(-4.5659) | 5 | 0.9988 | [28] |
|  |  | 2,3-Butanediol | (0.2151)-(1.6372) | (-5.9522)-(-4.4482) | 5 | 0.9997 | [28] |
|  |  | Diethylene glycol | (0.2029)-(1.6448) | (-5.9915)-(-4.4482) | 5 | 0.9997 | [29] |
|  |  | Furfuryl alcohol | (-0.2046)-(1.767) | (-6.0748)-(-4.1997) | 6 | 0.9989 | [32] |
|  |  | Guaiacol | (-0.4604)-(1.6799) | (-6.5713)-(-4.3428) | 6 | 0.9993 | [30] |
|  |  | Levulinic acid | (-0.2132)-(1.7573) | (-5.9145)-(-3.8538) | 6 | 0.9998 | [32] |
|  |  | Phenol | (0.2103)-(1.6363) | (-5.843)-(-4.3051) | 5 | 0.9998 | [29] |
|  |  | Triethylene glycol | (0.1756)-(1.6409) | (-5.684)-(-4.0984) | 5 | 0.9996 | [29] |
|  |  | Regardless of the effect of changing HBD structures | (-0.4604)-(1.767) | (-6.5713)-(-3.8538) | 48 | 0.8918 | [28-30, 32] |
|  |  |  |  |  |  |  |  |
| 323 | 1:4 | 1,2-Propanediol | (0.2029)-(1.6483) | (-6.2146)-(-4.733) | 5 | 0.9985 | [28] |
|  |  | 1,4-Butanediol | (0.207)-(1.6467) | (-6.1193)-(-4.5756) | 5 | 0.9986 | [28] |
|  |  | 2,3-Butanediol | (0.1519)-(1.6341) | (-5.8091)-(-4.3275) | 5 | 0.9984 | [28] |
|  |  | Diethylene glycol | (0.1544)-(1.6618) | (-6.0748)-(-4.4063) | 5 | 0.9992 | [29] |
|  |  | Furfuryl alcohol | (-0.4277)-(1.7331) | (-6.1658)-(-4.0923) | 6 | 0.9982 | [32] |
|  |  | Guaiacol | (-0.6033)-(1.6777) | (-6.5713)-(-4.2475) | 6 | 0.9999 | [30] |
|  |  | Levulinic acid | (-0.1863)-(1.7651) | (-5.7446)-(-3.755) | 6 | 0.9999 | [32] |
|  |  | Phenol | (0.1672)-(1.666) | (-5.9145)-(-4.3125) | 5 | 0.998 | [29] |
|  |  | Triethylene glycol | (0.1723)-(1.6492) | (-5.7138)-(-4.0745) | 5 | 0.9997 | [29] |
|  |  | regardless of the effect of changing HBD structures | (-0.6033)-(1.7651) | (-6.5713)-(-3.755) | 48 | 0.8591 | [28-30, 32] |

Figure S 1: Relationship between ln(x) and ln(P) in constant temperature 313 K for choline chloride/urea in 1:1.5, 1:2 and 1:2.5 molar ratio of HBA to HBD. [33]

|  |
| --- |

Figure S 2: linear relationship between ln(P) and ln(x) for each HBD involved in dataset no. (5) for instance.

|  |
| --- |

Figure S 3: Linear relationship between ln(x) and T in constant pressure about 3 bar and molar ratio equal to 1:3.

Figure S 4: Predicted values of x versus experimental values (a), Residuals versus experimental values (b), Standard residuals versus leverage (c) for unfixed temperatures dataset, dataset no. 1 (Eq. (7))

Figure S 5: Predicted values of x versus experimental values (a), Residuals versus experimental values (b), Standard residuals versus leverage (c) for unfixed temperatures dataset, dataset no. 2 (Eq. (10))

Figure S 6: Predicted values of x versus experimental values (a), Residuals versus experimental values (b), Standard residuals versus leverage (c) for fixed temperature dataset, dataset no. 3 (Eq. (12))

Figure S 7: Predicted values of x versus experimental values (a), Residuals versus experimental values (b), Standard residuals versus leverage (c) for fixed temperature dataset, dataset no. 4 (Eq. (14))

Figure S 8: Predicted values of x versus experimental values (a), Residuals versus experimental values (b), Standard residuals versus leverage (c) for fixed temperature dataset, dataset no 5. (Eq. (16))

Figure S 9: Predicted values of x versus experimental values (a), Residuals versus experimental values (b), Standard residuals versus leverage (c) for fixed temperature dataset, dataset no. 6 (Eq. (18))

Figure S 10: Predicted values of x versus experimental values (a), Residuals versus experimental values (b), Standard residuals versus leverage (c) for fixed temperature dataset, dataset no. 7 (Eq. (20))

Figure S 11: Predicted values of x versus experimental values (a), Residuals versus experimental values (b), Standard residuals versus leverage (c) for fixed temperature dataset, dataset no. 8 (Eq. (22))

Figure S 12: Predicted values of x versus experimental values (a), Residuals versus experimental values (b), Standard residuals versus leverage (c) for fixed temperature dataset, dataset no. 9 (Eq. (24))

Figure S 13: Predicted values of x versus experimental values (a), Residuals versus experimental values (b), Standard residuals versus leverage (c) for fixed temperature dataset, dataset no. 10 (Eq. (26)).

|  |
| --- |
|  |

Figure S 14: Standard residuals versus leverage for (a) dataset no. (11)) using Eq. (7) and (b) dataset no. (12) Eq. (10).

|  |
| --- |
|  |
|  |
|  |
|  |
|  |

Figure S 15: R^2^ and Q^2^ for sub-datasets with (a) P=4 bar, T=303 and molar ratio 1:3. (b) P=4 bar, T=303 and molar ratio 1:4. (c) P=5 bar, T=313 and molar ratio 1:3. (d) P=5 bar, T=313 and molar ratio 1:4. (e) P=3 bar, T=323 and molar ratio 1:3. (f) P=3 bar, T=323 and molar ratio 1:4.

References:

[1] A. Khajeh, M. Shakourian-Fard, and K. Parvaneh, "Quantitative structure-property relationship for melting and freezing points of deep eutectic solvents," *Journal of Molecular Liquids,* vol. 321, p. 114744, 2021.

[2] T. Lemaoui *et al.*, "Quantitative structure properties relationship for deep eutectic solvents using Sσ-profile as molecular descriptors," *Journal of Molecular Liquids,* vol. 309, p. 113165, 2020.

[3] Y. Benguerba, I. M. Alnashef, A. Erto, M. Balsamo, and B. Ernst, "A quantitative prediction of the viscosity of amine based DESs using Sσ-profile molecular descriptors," *Journal of Molecular Structure,* vol. 1184, pp. 357-363, 2019.

[4] T. Lemaoui *et al.*, "Prediction of electrical conductivity of deep eutectic solvents using COSMO-RS sigma profiles as molecular descriptors: a quantitative structure–property relationship study," *Industrial & Engineering Chemistry Research,* vol. 59, no. 29, pp. 13343-13354, 2020.

[5] A. K. Halder, R. Haghbakhsh, I. V. Voroshylova, A. R. C. Duarte, and M. N. D. Cordeiro, "Predicting the Surface Tension of Deep Eutectic Solvents: A Step Forward in the Use of Greener Solvents," *Molecules,* vol. 27, no. 15, p. 4896, 2022.

[6] F. Mjalli, K. Shahbaz, M. Hashim, and I. AlNashef, "Surface tension of ionic liquids analogues using the QSPR correlation," *International Journal of Chemical Engineering and Applications,* vol. 4, no. 3, p. 96, 2013.

[7] K. A. Kurnia, M. Zunita, J. A. Coutinho, I. G. Wenten, and D. Santoso, "Development of quantitative structure-property relationship to predict the viscosity of deep eutectic solvent for CO2 capture using molecular descriptor," *Journal of Molecular Liquids,* vol. 347, p. 118239, 2022.

[8] A. K. Halder, P. Ambure, Y. Perez-Castillo, and M. N. D. Cordeiro, "Turning deep-eutectic solvents into value-added products for CO2 capture: A desirability-based virtual screening study," *Journal of CO2 Utilization,* vol. 58, p. 101926, 2022.

[9] O. Alioui, Y. Benguerba, and I. M. Alnashef, "Investigation of the CO2-solubility in deep eutectic solvents using COSMO-RS and molecular dynamics methods," *Journal of Molecular Liquids,* vol. 307, p. 113005, 2020.

[10] S. Sarmad, D. Nikjoo, and J.-P. Mikkola, "Amine functionalized deep eutectic solvent for CO2 capture: Measurements and modeling," *Journal of Molecular Liquids,* vol. 309, p. 113159, 2020.

[11] M. B. Haider and R. Kumar, "Solubility of CO2 and CH4 in sterically hindered amine-based deep eutectic solvents," *Separation and Purification Technology,* vol. 248, p. 117055, 2020.

[12] H. S. Salehi, R. Hens, O. A. Moultos, and T. J. Vlugt, "Computation of gas solubilities in choline chloride urea and choline chloride ethylene glycol deep eutectic solvents using Monte Carlo simulations," *Journal of Molecular Liquids,* vol. 316, p. 113729, 2020.

[13] M. B. Haider, D. Jha, B. M. Sivagnanam, and R. Kumar, "Modelling and simulation of CO2 removal from shale gas using deep eutectic solvents," *Journal of Environmental Chemical Engineering,* vol. 7, no. 1, p. 102747, 2019.

[14] N. R. Mirza, N. J. Nicholas, Y. Wu, K. A. Mumford, S. E. Kentish, and G. W. Stevens, "Experiments and thermodynamic modeling of the solubility of carbon dioxide in three different deep eutectic solvents (DESs)," *Journal of Chemical & Engineering Data,* vol. 60, no. 11, pp. 3246-3252, 2015.

[15] R. Haghbakhsh and S. Raeissi, "Modeling the phase behavior of carbon dioxide solubility in deep eutectic solvents with the cubic plus association equation of state," *Journal of Chemical & Engineering Data,* vol. 63, no. 4, pp. 897-906, 2017.

[16] T. Altamash *et al.*, "Gas solubility and rheological behavior of natural deep eutectic solvents (NADES) via combined experimental and molecular simulation techniques," *ChemistrySelect,* vol. 2, no. 24, pp. 7278-7295, 2017.

[17] C. H. Dietz *et al.*, "PC-SAFT modeling of CO2 solubilities in hydrophobic deep eutectic solvents," *Fluid Phase Equilibria,* vol. 448, pp. 94-98, 2017.

[18] A. Kamgar, S. Mohsenpour, and F. Esmaeilzadeh, "Solubility prediction of CO2, CH4, H2, CO and N2 in Choline Chloride/Urea as a eutectic solvent using NRTL and COSMO-RS models," *Journal of Molecular Liquids,* vol. 247, pp. 70-74, 2017.

[19] L. F. Zubeir, C. Held, G. Sadowski, and M. C. Kroon, "PC-SAFT modeling of CO2 solubilities in deep eutectic solvents," *The Journal of Physical Chemistry B,* vol. 120, no. 9, pp. 2300-2310, 2016.

[20] R. Ullah *et al.*, "A detailed study of cholinium chloride and levulinic acid deep eutectic solvent system for CO 2 capture via experimental and molecular simulation approaches," *Physical Chemistry Chemical Physics,* vol. 17, no. 32, pp. 20941-20960, 2015.

[21] G. García, M. Atilhan, and S. Aparicio, "A theoretical study on mitigation of CO2 through advanced deep eutectic solvents," *International Journal of Greenhouse Gas Control,* vol. 39, pp. 62-73, 2015.

[22] G. Garcia, M. Atilhan, and S. Aparicio, "Interfacial properties of deep eutectic solvents regarding to CO2 capture," *The Journal of Physical Chemistry C,* vol. 119, no. 37, pp. 21413-21425, 2015.

[23] E. Ali *et al.*, "Solubility of CO2 in deep eutectic solvents: experiments and modelling using the Peng–Robinson equation of state," *Chemical Engineering Research and Design,* vol. 92, no. 10, pp. 1898-1906, 2014.

[24] Y. Xie, H. Dong, S. Zhang, X. Lu, and X. Ji, "Solubilities of CO2, CH4, H2, CO and N2 in choline chloride/urea," *Green Energy & Environment,* vol. 1, no. 3, pp. 195-200, 2016.

[25] Y. Liu *et al.*, "Screening deep eutectic solvents for CO2 capture with COSMO-RS," *Frontiers in chemistry,* vol. 8, p. 82, 2020.

[26] J. Wang, Z. Song, L. Chen, T. Xu, L. Deng, and Z. Qi, "Prediction of CO2 solubility in deep eutectic solvents using random forest model based on COSMO-RS-derived descriptors," *Green Chemical Engineering,* vol. 2, no. 4, pp. 431-440, 2021.

[27] P. Kumar, A. Kumar, J. Sindhu, and S. Lal, "Quasi-SMILES as a basis for the development of QSPR models to predict the CO2 capture capacity of deep eutectic solvents using correlation intensity index and consensus modelling," *Fuel,* vol. 345, p. 128237, 2023.

[28] Y. Chen, N. Ai, G. Li, H. Shan, Y. Cui, and D. Deng, "Solubilities of carbon dioxide in eutectic mixtures of choline chloride and dihydric alcohols," *Journal of Chemical & Engineering Data,* vol. 59, no. 4, pp. 1247-1253, 2014.

[29] G. Li, D. Deng, Y. Chen, H. Shan, and N. Ai, "Solubilities and thermodynamic properties of CO2 in choline-chloride based deep eutectic solvents," *The Journal of Chemical Thermodynamics,* vol. 75, pp. 58-62, 2014.

[30] X. Liu, B. Gao, Y. Jiang, N. Ai, and D. Deng, "Solubilities and thermodynamic properties of carbon dioxide in guaiacol-based deep eutectic solvents," *Journal of Chemical & Engineering Data,* vol. 62, no. 4, pp. 1448-1455, 2017.

[31] M. B. Haider, D. Jha, B. Marriyappan Sivagnanam, and R. Kumar, "Thermodynamic and kinetic studies of CO2 capture by glycol and amine-based deep eutectic solvents," *Journal of Chemical & Engineering Data,* vol. 63, no. 8, pp. 2671-2680, 2018.

[32] M. Lu, G. Han, Y. Jiang, X. Zhang, D. Deng, and N. Ai, "Solubilities of carbon dioxide in the eutectic mixture of levulinic acid (or furfuryl alcohol) and choline chloride," *The Journal of Chemical Thermodynamics,* vol. 88, pp. 72-77, 2015.

[33] X. Li, M. Hou, B. Han, X. Wang, and L. Zou, "Solubility of CO2 in a choline chloride+ urea eutectic mixture," *Journal of Chemical & Engineering Data,* vol. 53, no. 2, pp. 548-550, 2008.
